# Assessing Motor Performance in Preschool Children: The Zurich Neuromotor Assessment-2 and the Movement Assessment Battery for Children-2

**DOI:** 10.1177/00315125211025246

**Published:** 2021-06-15

**Authors:** Gerda van der Veer, Erica Kamphorst, Alexander Minnaert, Marja Cantell, Tanja H. Kakebeeke, Suzanne Houwen

**Affiliations:** 1 Inclusive and Special Needs Education Unit, Faculty of Behavioural and Social Sciences, University of Groningen, Groningen, the Netherlands; 2 Child Development Center, University Children’s Hospital Zürich, Zürich, Switzerland

**Keywords:** Zurich Neuromotor Assessment-2, Movement Assessment Battery for Children-2, motor skills, motor abilities, early childhood, psychometric properties

## Abstract

Comparing motor assessment tools that are available for young children is important in order to select the most appropriate clinical and research tools. Hence, this study compared motor performance assessed with the Zurich Neuromotor Assessment-2 (ZNA-2) to the Movement Assessment Battery for Children-2 (MABC-2). The sample consisted of 169 children, aged 3–5 years (87 boys; 51%). We used Pearson correlations to examine relationships between the ZNA-2 and MABC-2 component and total scores. In addition, Pearson correlations were performed between individual fine motor and balance items of the ZNA-2 and MABC-2. Results were that the total scores of the ZNA-2 and MABC-2 correlated moderately (*r* = .40, *p* < .001). Non-significant to moderate correlations were found between components (*r = −*.00 to .47) and between individual items of fine motor skills (*r* = .04 to .38) and balance (*r* = −.12 to .38). Thus, the ZNA-2 and MABC-2 measure partly similar and partly different aspects of motor performance.

## Introduction

Three to five-year old children experience a remarkable pace and extent of development in their motor repertoire. The attainment of basic motor skills, such as balance, locomotion, and object control, in this age period lays the foundation for learning more complex motor skills needed for daily life and physical activities (Gabbard, 2018; Piek et al., 2012). Practicing various motor skills also contributes to the development of cognitive and social skills (Diamond, 2007). More specifically, the expanding range of motor skills provides multiple opportunities for young children to engage in environmental exploration, perceptual learning, and social interaction – skills that match children’s increasing need for autonomy (Oudgenoeg-Paz et al., 2015; Walle & Campos, 2014). With the acknowledgement that motor skills are linked to cognitive and social-emotional skills, the assessment of motor performance in 3–5-year-old children has gained importance (Piek et al., 2012).

There are several assessment tools available to examine motor performance at the preschool age (Piek et al., 2012). One of the most frequently used tests by health care professionals in Europe for this purpose in (young) children is the Movement Assessment Battery for Children-2 (MABC-2; Henderson et al., 2007). The MABC-2 is a validated and norm-referenced test to detect motor coordination difficulties in children aged 3–16 years, and it has been recommended for the detection of Developmental Coordination Disorder (Blank et al., 2019). But the MABC-2 may be susceptible to ceiling effects for better performers (French et al., 2018; Kokštejn et al., 2018). Ceiling effects make a test less suitable for assessing motor performance in typically developing children, as differentiation between above average performers cannot be done (Cools et al., 2009; de Niet et al., 2021). Some health care professionals may therefore use the norm-referenced Zurich Neuromotor Assessment-2 (ZNA-2; Kakebeeke et al., 2019). In contrast to the MABC-2, the ZNA-2 battery has been designed to assess the entire range of a child’s motor performance (i.e., from very poor to very good) for children aged 3 - 18 years (Kakebeeke et al., 2019). Despite this difference, unlike other motor tests, such as the Bruininks-Oseretsky Test of Motor Proficiency Second Edition (Bruininks & Bruininks, 2005), the Körperkoordinationstest für Kinder (Kiphard & Schilling, 2007), and the Test of Gross Motor Development-3 (Ulrich, 2013), the ZNA-2 and MABC-2 cover both the assessment of fine and gross motor skills over the 3-5 year age range. To permit practitioners and researchers to compare the test results of individual 3-5-year-old children who may have been evaluated with these two instruments, or even the results of any child who might have been given one of these tools on one instance and the other later, it is important to empirically compare and establish performances on these two different motor assessment tools to better understand how they may be related.

It is important to note that motor performance is a concept covering a variety of terms (e.g., motor skills, motor abilities) used to describe goal-directed human movement (Burton & Miller, 1998). Although all these different terms are related, they describe conceptually distinct constructs. Motor skills can be seen as the qualitative expression of a specific movement pattern (e.g., hop, catch), while motor abilities can be viewed as general traits or capacities that underlie the performance of a variety of motor skills (Burton & Miller, 1998). A similarity between the ZNA-2 and MABC-2 is that they are both, to some extent, based on the normative functional approach (Wilson, 2005); that is, they both yield a quantitative measure (e.g., response time, proficiency rating, and accuracy scores) that indicates performance level (norm-referenced), and both focus on functional motor skills. In addition, the ZNA-2 also includes tasks representing motor abilities (Kakebeeke et al., 2018), which reflect internal neurological and neuromotor processes and thus provide information about the neurological basis of a child’s motor functioning (Burton & Miller, 1998). Thus, the ZNA-2 and MABC-2 may provide different information about a child’s motor performance. Neither assessment tool necessarily claims to measure the same aspects of motor performance; however, researchers often use each tool in an attempt to answer similar clinical and research questions. Thus, there is a need to determine how seemingly similar and/or different aspects of motor performance included in the two assessment tools are or are not related (Logan et al., 2014; Scheuer et al., 2019).

A study comparing an earlier version of the ZNA for young children (ZNA3-5) with the MABC-2 revealed a rank correlation of .77 between the total scores of the two tests, and rank correlations higher than .50 between their components, making the tests seemingly comparable in their face validity (Kakebeeke et al., 2016). In addition, components measuring different motor constructs correlated within a range from .31-.52. Thus, some components measure similar constructs, while others assess different aspects of motor performance. Given the more recent development of an updated version of the ZNA, the ZNA-2, a renewed comparison between the MABC-2 and this updated version of the ZNA-2 is both necessary and timely. The aim of the present study was to compare the motor performance of 3-5-year-old children on the ZNA-2 and MABC-2.

## Method

### Participants

The participant sample for this study was drawn from a larger research project ‘MELLE’ (Motor skills, Executive functions, Language, and LEarning outcomes in preschool children; see also Houwen et al., 2019), in which 3–5-year-old children are being followed intensively through their development with respect to their motor skills, executive functions, and language abilities. The MELLE-study protocol received approval by the Ethics Review Committee of the Department of Pedagogical and Educational Sciences, Faculty of Behavioural and Social Sciences, University of Groningen, and parents of all the child participants gave their written informed consent for data from their children’s evaluations to be used in research, in accordance with the Declaration of Helsinki.

From March 2016 to December 2018, a sample of 193 Dutch preschool children was recruited from preschools, kindergartens, day-care centers, and primary schools in the north of the Netherlands. Additionally, social media, mouth-to-mouth recruitment, and flyers and posters in supermarkets, stores, and playgrounds were used to recruit participants. Participant inclusion criteria were: (a) aged between 36 and 72 months; (b) no signs of a medical condition (e.g., heart disease), neurological disorder (e.g., cerebral palsy), or intellectual or physical disability (e.g., club foot); (c) normal hearing and normal or corrected to normal visual sight; (d) being able to follow test instructions; and (e) having parents/caretakers with sufficient proficiency in written Dutch to be able to complete the questionnaires. The inclusion criteria were checked by phone interview. In addition, parents filled out a sociodemographic questionnaire. Twenty-four children were excluded from the sample for unreliable assessments on one or both motor tests due to refusal, lack of concentration, or lack of motivation. The final sample consisted of 169 children (87 boys; 51%), aged 36 to 71 months old (*M* = 50.8 months, *SD* = 10.1 months). The group of 3-year-olds consisted of 35 boys and 37 girls (age range 36 to 47 months), the group of 4-year-olds consisted of 30 boys and 24 girls (age range 48 to 59 months), and the group of 5-year-olds consisted of 22 boys and 21 girls (age range 60 to 71 months).

### Instruments

*Zurich Neuromotor Assessment Second Edition (ZNA-2)*. The ZNA-2 (Kakebeeke et al., 2019) is a standardized test that aims to describe the development of neuromotor functioning in 3–18-year-old children. Motor skills and motor abilities are quantitatively examined by measuring the following components: fine motor skills (pegboard, bolts, and beads), pure motor skills (repetitive hand, foot, and finger movements, alternating hand and foot movements, and sequential finger movements), static balance (one-leg stand with eyes open and with eyes closed), and dynamic balance (jumping sidewards, chair-rise, and standing long jump). All items are measured in seconds; only the long jump item is measured in centimeters. Age- and gender-based standard scores (z-scores) can be calculated for the components and the total score. Norm reference values are available for Swiss children (Kakebeeke et al., 2018), and these were used to calculate the motor performance of the study sample.

The ZNA-2 has shown moderate to good test-retest reliabilities for its components, with ICC’s ranging from .67 to .84 in a sample of Swiss children aged 3–18 years. Intra-rater reliabilities were excellent with ICC’s of 1.00. Inter-rater reliabilities were also excellent with ICC’s of .92 and higher. A factorial study on the original ZNA, of which the item groupings of the ZNA-2 are based, showed good factorial validity (Rousson & Gasser, 2004). A comparison study between the ZNA 3-5 (an earlier version of the ZNA for young children) and the MABC-2 supported convergent validity (Kakebeeke et al., 2016). Discriminant validity has not yet been investigated.

*Movement Assessment Battery for Children Second Edition (MABC-2).* The MABC-2 (Dutch version; Henderson et al., 2010) is a norm-referenced test that aims to detect motor impairment in children aged 3–16 years. It consists of three age bands, which have specific scoring and task variations. Age band 1 (3–6 years) was used in the present study. The MABC-2 includes eight tasks measuring three components: manual dexterity (‘Posting coins’, ‘Threading beads’, and ‘Drawing trail’), aiming and catching (‘Catching a bean bag’ and ‘Throwing a beanbag onto a mat’), and balance (‘One-leg stand’, ‘Walking heels raised’, and ‘Jumping on mats’). The posting coins, threading beads and one-leg stand tasks are measured in seconds. Catching a beanbag, throwing a beanbag onto a mat, walking heels raised, and jumping on mats are measured in number of successful jumps. The drawing trail task is measured in number of errors. Age-based standard scores (range 1–19, *M* = 10, *SD* = 3) and percentile scores can be calculated for the three components and for a total score. The MABC-2 has been normed for Dutch children. The psychometric properties of age band 1 of the MABC-2 suggest that it is a valid and reliable measure to be used in preschoolers (Ellinoudis et al., 2011; Psotta & Brom, 2016; Smits-Engelsman et al., 2011). A comparison study between age band 1 of the MABC-2 and the Peabody Developmental Motor Scales–2 supported convergent validity in preschool children (Hua et al., 2013). To our knowledge, discriminant validity of age band 1 of the MABC-2 has not been investigated yet.

### Procedure

Test administrators were graduate students from Pedagogical and Educational Sciences, Psychology or Human Movement Sciences and were all trained intensively to ensure reliable test results. The training process consisted of reading test manuals, practicing on each other with the tests, administering two videotaped practice assessments which were evaluated during group sessions, and scoring the performance of the children during the practice assessments. Students were provided individual feedback on their practice assessments, and, above and beyond, had to pass their training before administering any formal assessments.

The data necessary for this study were gathered in two sessions, each lasting up to 90–120 minutes; in the first session, the children performed the MABC-2-NL, and in the second session, the ZNA-2. Next to the ZNA-2 and MABC-2, children completed a battery of cognitive and language tests as part of the MELLE-project. The assessments were videotaped for scoring purposes. Children received stickers after every task as encouragement. When necessary, short individually tailored breaks were used to maintain motivation and attention. When testing was completed children were given a small gift (<2 euro) and a certificate for their participation in the MELLE-project. Parents received a report of their child’s test results. Participant data were coded and stored using a personalized study identifier to ensure confidentiality.

### Statistical Analysis

Data were analyzed using SPSS version 25.0. Descriptive statistics (frequencies, means, and standard deviations) were calculated to characterize the motor performance of the sample on all ZNA-2 and MABC-2 components and total scores. The rate of missing test data varied from 0% (age and gender) to 52.1% (ZNA-2 total score) (see Online Appendix A for a more detailed overview of the missing values per variable). Little’s MCAR test indicated that the missing values of the motor components and total scores (Little’s MCAR test: χ^2^ (156) = 177.634, *p* = .113) as well as the missing values of the individual items (Little’s MCAR test: χ^2^ (488) = 484.113, *p* = .541) were missing on a completely random basis. To account for potential bias resulting from missing data and to increase statistical power, we performed multiple imputation with full conditional specification. Multiple imputation is a sophisticated and flexible routine for handling missing data. Several studies have shown that accuracy and reliability of multiple imputation is attainable, even with substantial proportions of missing data (Graham & Schafer, 1999; Madley-Dowd et al., 2019). Regarding relationships between components of the ZNA-2 and MABC-2, we performed multiple imputation using the components and total scores as both predictors and variables to be imputed. Age and gender were used as predictors. Information of individual items were used so as to be able to impute more reliably by constructing variables of the mean of individual items per component. Regarding relationships between individual items of fine motor skills and balance of both motor tests, the following MABC-2 items were imputed and used as predictors: ‘Posting coins dominant (d) hand’, ‘Posting coins nondominant (nd) hand’, ‘Threading beads’, ‘Drawing trail’, ‘One-leg stand’, ‘Walking heels raised’, and ‘Jumping on mats’. The following ZNA-2 items were imputed and used as predictors: ‘Pegboard (d)’, ‘Pegboard (nd)’, ‘Bolts (d)’, ‘Bolts (nd)’, ‘Beads’, static balance component, ‘Jumping sidewards’, ‘Chair-rise’, and ‘Standing long jump’. The aiming and catching items of the MABC-2, the pure motor items of the ZNA-2, age, and gender, were used as predictors. For both imputation models, twenty multiple imputed data sets were created. All subsequent analyses were conducted on each imputed data set individually, and the results were pooled.

The degree of association between the ZNA-2 and MABC-2 components and total scores was assessed by bivariate Pearson correlation analyses. Next, we analyzed the relationship between the ZNA-2 and MABC-2 more in-depth by performing bivariate Pearson correlation analyses between individual items of components with theoretically similar motor constructs (i.e., fine motor and balance items). Based on Kakebeeke et al. (2016), we expected mostly moderate (>.30) to strong (>.50) effect sizes. An a priori power analysis (G*Power Version 3.1.9.2; Faul et al., 2009) revealed that our sample size of 160 was sufficient to detect correlations as low as .20 (with a power of .80 and alpha level of .05). Examination of histograms of all imputed data sets showed that the assumption of normality was not violated. In addition, no serious violations of linearity and outliers were observed when boxplots and scatterplots of all imputed data sets were analyzed.

## Results

### Descriptive

An overview of the descriptive data for the components, total scores, and individual test items of the ZNA-2 and MABC-2 is provided in Table 1 (see Online Appendix B for a more detailed overview of these test characteristics per participant age). Cohen’s *d* analysis showed that the original and pooled means of the components, total scores, and individual items were similar for the whole sample, except for the component pure motor skills of the ZNA-2, which differed weakly. For 3-year-old children, there were small differences between the original and pooled means of the individual item ‘Jumping sidewards’. For 5-year-olds, there were small differences between the original and pooled means of the component static balance of the ZNA-2 and the ZNA-2 total score. There were no differences between the original and pooled means for children aged four years.

**Table 1.** Descriptives on the ZNA-2 and the MABC-2.

	Original			Pooled			
	*n*	*M*	*SD*	*n*	*M*	*SD*	Effect size
*ZNA-2*							
Individual items							
Pegboard (d)	163	.18	.98	169	.17	0.99	.00
Pegboard (nd)	162	.04	1.14	169	.03	1.16	.01
Bolts (d)	163	.75	1.04	169	.76	1.05	−.01
Bolts (nd)	160	.56	1.01	169	.56	1.02	.00
Beads	165	.21	1.02	169	.21	1.02	.00
Repetitive foot (d)^a^	162	−.33	1.28				
Repetitive foot (nd)^a^	160	−.39	1.36				
Alternating foot (d)^a^	159	−.16	1.06				
Alternating foot (nd)^a^	159	−.03	.96				
Repetitive hand (d)^a^	162	−.70	1.52				
Repetitive hand (nd)^a^	157	−.70	1.40				
Alternating hand (d)^a^	158	−.37	1.12				
Alternating hand (nd)^a^	158	−.31	1.25				
Repetitive fingers (d)^a^	160	−.40	1.57				
Repetitive fingers (nd)^a^	159	−.31	1.57				
Sequential fingers (d)^a^	149	.30	1.05				
Sequential fingers (nd)^a^	147	.24	1.01				
Jump sideways	153	−.05	.74	169	−.05	0.75	.00
Chair rise	155	−.46	1.34	169	−.42	1.37	−.03
Long jump	152	−.11	1.27	169	−.12	1.29	.01
Components							
Fine motor skills	148	.55	1.01	169	.49	1.01	−.06
Pure motor skills	114	−.26	1.36	169	−.45	.96	−.20
Static balance	153	−.38	1.06	169	−.37	1.11	.01
Dynamic balance	128	−.31	1.10	169	−.31	1.18	.00
Total score	81	−.26	1.04	169	−.23	1.04	.03
*MABC-2*							
Individual items							
Posting coins (d)	167	10.63	2.56	169	10.63	2.56	.00
Posting coins (nd)	165	10.58	2.46	169	10.55	2.47	.01
Beads	166	10.17	2.42	169	10.17	2.43	.00
Drawing trail	168	9.21	2.48	169	9.20	2.49	.00
Catching a bean bag^a^	164	8.31	2.35				
Throwing a bean bag^a^	162	9.75	3.06				
One-leg stand	166	9.66	3.03	169	9.66	2.36	.00
Walking on toes	167	10.63	2.56	169	10.63	3.07	.00
Jumping on mats	165	10.58	2.46	169	10.55	3.04	.01
Components							
Manual dexterity	164	10.11	2.85	169	10.07	2.85	−.01
Aiming & catching	165	9.87	3.00	169	9.90	2.98	.01
Balance	159	8.99	3.06	169	9.02	3.07	.01
Total score	152	9.70	3.25	169	9.66	3.16	−.01

*Note.*^a^Pooled descriptives are not available for these items, because they were not multiply imputed. d: dominant hand; nd: nondominant hand.

### The Relationship Between Components and Total Scores of the ZNA-2 and MABC-2

Table 2 illustrates the bivariate correlations between the ZNA-2 and MABC-2 components and total scores. The total scores correlated moderately with each other (*r* = .40, *p* < .001). On component level, the highest correlations were observed between the fine motor skills component of the ZNA-2 and the manual dexterity component of the MABC-2 (*r* = .47, *p* < .001), and between the static balance component of the ZNA-2 and the balance component of the MABC-2 (*r* = .35, *p* < .001). The manual dexterity component of the MABC-2 correlated weakly to moderately with the other components and total score of the ZNA-2 (*r* = .18 to .37), with the exception of dynamic balance. The balance component of the MABC-2 correlated weakly to moderately with all ZNA-2 components and the total score (*r* = .17 to .39). There were no significant correlations between the aiming and catching components of the MABC-2 and the ZNA-2 components.

**Table 2.** Pearson Correlations Between the ZNA-2 and the MABC-2 Subtests and Total Scores.

	MABC-2			
ZNA-2	Manual dexterity	Aiming and catching	Balance	MABC-2 total score
Fine motor skills	.47**	.15	.24**	.41**
Pure motor skills	.18*	−.00	.20*	.16*
Static balance	.22**	.13	.35**	.34**
Dynamic balance	.06	.04	.17*	.12
ZNA-2 total score	.37**	.11	.39**	.40**

*Note.* *p < .05; **p < .01.

### The Relationship Between Individual Fine and Balance Items of the ZNA-2 and MABC-2

From Table 3 it is clear that mainly weak to moderate correlations were found between fine motor items of the ZNA-2 and MABC-2. The highest correlations were observed between ‘Pegboard (nd)’ and ‘Posting coins (nd)’ (*r* = .41, *p* < .001), between both beads items (*r* = .38, *p* < .001), between ‘Pegboard (d)’ and ‘Posting coins (d)’ (*r* = .32, *p* < .001), and between ‘Pegboard (nd)’ and ‘Beads’ of the MABC-2 (*r* = .30, *p* < .001). The lowest correlations were detected between the ‘Drawing trail’ and the ZNA-2 fine motor items (*r* = .04 to .16).

**Table 3.** Pearson Correlations Between Fine Motor Items of the ZNA-2 and MABC-2.

	MABC-2			
ZNA-2	Posting coins (d)	Posting coins (nd)	Beads	Drawing trail
Pegboard (d)	.32**	.29**	.27**	.12
Pegboard (nd)	.28**	.41**	.30**	.14
Bolts (d)	.19*	.19**	.14	.04
Bolts (nd)	.24**	.28**	.22**	.10
Beads	.24**	.27**	.38**	.16*

*Note.* d: dominant hand; nd: nondominant hand. *p < .05; **p < .01.

Table 4 shows the correlations between the individual balance items of the ZNA-2 and MABC-2. The highest correlation was found between ‘One-leg stand’ of the ZNA-2 and the ‘One-leg stand’ of the MABC-2 (*r* = .38, *p* < .001). Weak correlations were observed between ‘One-leg stand’ of the ZNA-2 and ‘Walking on toes’ of the MABC-2 (*r* = .21, *p* = .009) and between ‘Jumping sideways’ of the ZNA-2 and ‘One-leg stand’ of the MABC-2 (*r* = .23, *p* = .003). No significant correlations were found between the other balance items.

**Table 4.** Pearson Correlations Between Balance Items of the ZNA-2 and MABC-2.

	MABC-2		
ZNA-2	One-leg stand	Walking on toes	Jumping on mats
Static balance	.38**	.21**	.05
Jumping sideways	.23**	.03	.15
Chair rise	−.03	.01	.10
Standing long jump	.09	.13	−.12

*Note.* *p < .05; **p < .01.

## Discussion

The aim of this study was to compare the measured motor performance of 3–5-year-old children on the ZNA-2 and MABC-2. In general, we found a moderate relationship between the total scores of the ZNA-2 and MABC-2. Relationships between motor skill components of the ZNA-2 and MABC-2 varied from non-significant to moderate. As expected, the relationships between pure motor skills of the ZNA-2, measuring motor abilities, and the MABC-2 components, measuring motor skills, were non-significant to weak.

Our findings differed from Kakebeeke et al. (2016) in which the ZNA3-5 (an earlier version of the ZNA for young children) was compared to the MABC-2. Kakebeeke et al. (2016) showed higher correlations between components and total scores of the ZNA3-5 and MABC-2 (correlation between total scores .77) than the current study (correlation between total scores .40). Furthermore, Kakebeeke et al. (2016) found moderate-to-strong relationships between the components, while we found weak-to-moderate relationships. In line with Kakebeeke et al. (2016), relationships between the aiming and catching component of the MABC-2 and the ZNA-2 components were lowest. In a practical hindsight, the Swiss and Dutch studies took place in two different settings, at childcare center vs. at home, thus providing a different context for the assessments, yet there does not seem to be any simple explanation for the different correlation results. Perhaps future studies comparing motor tests in young children, such as the ZNA-2 and MABC-2, could include several levels of analysis incorporating task analysis with the context and child factors (such as executive functioning and temperament).

Examining in more detail the relationships between individual items of the components that intend to measure similar underlying motor constructs, the strength of the relationships between individual item seem to depend on the similarity of the test items. The relationships between similar items of both tests (i.e., beads and one-leg stand items) were moderate, while the relationships between seemingly different items (e.g., ‘Drawing trail’ of the MABC-2 with the fine motor items of the ZNA-2) were non-significant to weak. Thus our findings indicate that the ZNA-2 and MABC-2 measure motor performance in partly similar and partly different ways, revealing partly different underlying motor constructs in these two measures.

The low to modest relationships found between the ZNA-2 and MABC-2, despite some items that seemed similar at face value, may be explained by different task demands imposed on the child. Stemming from the theory of constraints, the production of movement is influenced by characteristics of the individual, the environment, and the task (Adolph & Hoch, 2019; Newell, 1986). Derived from this theory, differences in task demands were considered to be a reason why the associations were modest between items that at first sight seemed comparable. For example, both tests contain a manual dexterity task that included beads. The ‘Beads’ item of the ZNA-2 includes small round beads that need to be strung onto a thin thread. The beads are lying randomly in a hollow of the pegboard. After practice, in the test phase the child has one attempt to string five beads onto the thread as fast as possible. The ‘Threading beads’ item of the MABC-2 includes square beads and a thicker thread than in the ZNA-2. In addition, the setup is different from the ZNA-2 for which the beads are lying in a row and cannot easily roll off; and, after practice, in the ZNA-2 test phase the child has two attempts to string six beads onto the thread as quickly as possible. In sum, the disparities in the task demands of individual test items may cause some of the differences in motor performance, and subsequently, may explain modest associations.

Interestingly, differences in task demands also exist between the balance items. In the ‘One-leg stand’ of the MABC-2, the child is wearing gym shoes; but, in the ZNA-2, the child is not. In addition, in the ZNA-2 the child is requested to stand on one leg with eyes closed; whereas, in the MABC-2, the child is only asked to stand on one leg with eyes open. The ‘Chair-rise’ and ‘Jumping sidewards’ tasks from the ZNA-2 involve continuous movements in which a time element plays a role (Kakebeeke et al., 2019). ‘Walking on toes’ in the MABC-2 is a task that should be performed as accurately as possible without a time requirement and can thus be performed as slowly as needed. In the ‘Jumping on mats’ task from the MABC-2, 3- and 4-year-old children are allowed to complete the jumps in a discontinuous manner, and they can adjust their standing position on the mats (Henderson et al., 2007). Therefore, the performance on the balance tasks of the MABC-2 may be less dynamic than the dynamic balance tasks of the ZNA-2. Apart from all the above differences in test items and demands posed on the children, there are some parts of both tests for which there is no equivalent in the other test. Pencil skills and ball skills are only included in the MABC-2. The performance on these items are highly dependent on experience and practice (Hardy et al., 2012). Pure motor skills are only assessed with the ZNA-2 with the intention of examining the maturational state of the nervous system. Pure motor skills are skills that are not easily modified by experience or practice and are relatively stable across the lifespan (Burton & Miller, 1998; Cincotta & Ziemann, 2008; Mayston et al., 1999). Our results confirmed the notion that each assessment includes components that are unique for it and that are aimed at measuring specific motor constructs. In practice, although there is an overlap between the ZNA-2 and MABC-2, clinicians should choose a test or specific test components that targets the purpose of the assessment and provides the most useful information for that purpose.

The modest relationships found between the ZNA-2 and MABC-2 may also be partially explained by the large intra-individual variability in motor development, especially in early childhood (Adolph et al., 2015; Piek et al., 2012). In addition, early childhood is characterized by ordinary day-to-day variations in behavior, motivation, and attention that impact motor performance (Payne & Isaacs, 2016). Because of behavioral and performance instability, it is important not to base diagnostic conclusions in young children on assessment at one single time point, but to assess motor functioning at multiple times in order to examine the temporal stability of a young child’s developmental motor problems (Blank et al., 2019; Eldred & Darrah, 2010).

The modest relationships we found between the ZNA-2 and MABC-2 may also be partially due to the internal structure of one or both of these motor tests. Although we were not able to examine the internal structure of the tests due to our small sample size, both a three-factor structure (Psotta & Brom, 2016) and a one-factor structure of the MABC-2 (Okuda et al., 2019; Schulz et al., 2011; van der Veer et al., 2020) seem viable in preschool children. Furthermore, the factor structure of the original ZNA has been supported by Rousson and Gasser (2004). The ZNA-2 is likely to have a similar factorial structure, because the ZNA-2 is closely based on the original ZNA. Thou our findings may be considered together with the original ZNA factor structure. As such, we are confident that our study provides at least partial information on the relationship between both tests. Nevertheless, we cannot fully rule-out the possibility that our findings are limited to the performance profiles of the current sample.

### Limitations and Directions for Further Research

A few study limitations should be addressed when considering these results. Despite rigorous attempts to provide the best possible test administration for young children, these data contained quite a few missing values, mostly prevalent in the components of pure motor skills and dynamic balance on the ZNA-2. It is commonly known that assessing preschoolers is challenging, due to fluctuations in their behavior, attention, motivation and mood (Malina, 2004; Williams et al., 2019). The amount of missing data decreased with age, suggesting that the tasks are feasible in this age range but might need some extra attention and/or modification in test administration for the youngest in this age group. It is difficult to draw a conclusion on how the missing data might have affected the outcomes. A second limitation is that the instruments were administered on two different days. Although the interval between the MABC-2 and ZNA-2 was generally short, this time gap may have reduced measured agreement as a result of ordinary day-to-day variations in young children’s behavior and motor performance, as already mentioned above. Third, like in many other studies where children have been recruited from the community (e.g., Hoskens et al., 2018; Houwen et al., 2017), the sample was not fully representative with respect to the parents’ educational level. The sample consisted mainly of children with highly educated mothers. Fourth, our sample of young children did not include children with a formal diagnosis of Developmental Coordination Disorder (DCD). Due to the large intra-individual variability in early motor development (Darrah et al., 2009) and the lack of stability of (mild) motor coordination difficulties at a young age (Houwen et al., 2021; van Waelvelde et al., 2010), DCD is difficult to identify before the age of five years (Blank et al., 2019). Therefore, the European Academy of Childhood Disability does not recommend diagnosis of DCD before the age of five years (Blank et al., 2019). Finally, the small sample size did not allow analysis of the internal structures of the ZNA-2 and MABC-2. Subsequently, we could not examine if or how the internal structures of either or both of these motor tests might affect their relationship. Future research is recommended to examine the internal structures of both motor tests.

## Conclusion

Although both the ZNA-2 and MABC-2 focus on the general concept of motor performance, our findings indicate that they measure distinct aspects of motor performance. Accordingly, the ZNA-2 and MABC-2 may provide different and complementary information regarding a child’s motor performance. A relevant fact in this context is that the ZNA-2 and MABC-2 partly use similar motor tasks as test items, with differences arising only in contextual details such as in the ‘One-leg stand’. In addition, they assess similar skills with different motor tasks, such as the ‘Bolts’ task of the ZNA-2 and the ‘Drawing trail’ of the MABC-2 both measuring fine motor performance. Therefore, practitioners and researchers should be aware of the distinct differences in content and outcome measurements of these tools. In practice, this means that when the aim is to assess particular motor skills such as ball skills, then the MABC-2 is a good understandable choice. As a test, it enables the examiner to track intervention effects at motor skills, such as aiming and catching (Heus et al., 2020). As the ZNA-2 is less dependent on practice and is also focused on the development of motor abilities (Kakebeeke et al., 2021), the ZNA-2 may detect different children as having motor coordination difficulties than the MABC-2. Lastly, for some populations, such as young children at risk of motor difficulties, a careful task analysis may be an essential step in disentangling the everyday variability of motor performance from variability rising from neurological causes. Such information is imperative in early assessment procedures and for well-targeted and efficient intervention contents.

## Supplemental Material

sj-pdf-1-pms-10.1177_00315125211025246 - Supplemental material for Assessing Motor Performance in Preschool Children: The Zurich Neuromotor Assessment-2 and the Movement Assessment Battery for Children-2 Click here for additional data file. Supplemental material, sj-pdf-1-pms-10.1177_00315125211025246 for Assessing Motor Performance in Preschool Children: The Zurich Neuromotor Assessment-2 and the Movement Assessment Battery for Children-2 by Gerda van der Veer, Erica Kamphorst, Alexander Minnaert, Marja Cantell, Tanja H. Kakebeeke and Suzanne Houwen in Perceptual and Motor Skills
