# Supplementary material for: Assessing Motor Performance in Preschool Children: The Zurich Neuromotor Assessment-2 and the Movement Assessment Battery for Children-2
Source: Percept Mot Skills. 2021 Jun 15;128(5):2014–32. doi: 10.1177/00315125211025246 (PMC8414808; doi:10.1177/00315125211025246)
Supplement: sj-pdf-1-pms-10.1177_00315125211025246 - Supplemental material for Assessing Motor Performance in Preschool Children: The Zurich Neuromotor Assessment-2 and the Movement Assessment Battery for Children-2 [file sj-pdf-1-pms-10.1177_00315125211025246.pdf]

Appendix B

Table B.1

Descriptives on the ZNA-2 and the MABC-2 in 3-year old children

	Original			Pooled			Effect size
	n	*M*	*SD*	*n*	*M*	*SD*	
Pegboard (d)	69	.17	.99	72	.15	1.00	.02
Pegboard (nd)	67	.03	1.10	72	.00	1.14	.02
Bolts (d)	68	1.18	.93	72	1.18	.94	.00
Bolts (nd)	63	.87	.93	72	.84	.97	.04
Beads	68	.19	.89	72	.20	.90	-.01
Repetitive foot (d)*	69	-.55	1.37				
Repetitive foot (nd)*	66	-.81	1.50				
Alternating foot (d)*	65	-.41	.87				
Alternating foot (nd)*	65	-.18	.93				
Repetitive hand (d)*	68	-1.17	1.14				
Repetitive hand (nd)*	65	-1.00	1.26				
Alternating hand (d)*	66	-.50	.97				
Alternating hand (nd)*	64	-.75	.84				
Repetitive fingers (d)*	65	-1.03	1.61				
Repetitive fingers (nd)*	67	-.68	1.67				
Sequential fingers (d)*	58	.12	.62				
Sequential fingers (nd)*	58	.11	.63				

Jumping sideways	64	-.05	.04	72	-.06	.27	.38
Chair rise	64	-.87	1.29	72	-.85	1.32	-.02
Long jump	61	-.49	1.26	72	-.45	1.28	-.03
Fine motor skills	55	.83	.94	72	.66	.99	-.17
Pure motor skills	38	-.82	1.37	72	-.95	.91	-.14
Static balance	70	.00	.91	72	.00	.90	.00
Dynamic balance	49	-.68	.91	72	-.72	1.11	.04
Total score	27	-.25	1.05	72	-.37	.98	-.12
MABC-2							
Posting coins (d)	72	10.61	2.27				
Posting coins (nd)	71	10.83	2.69	72	10.79	2.70	.01
Beads	71	10.66	2.22	72	10.68	2.23	-.01
Drawing trail	72	10.01	2.50				
Catching a bean bag*	72	10.60	2.56				
Throwing a bean bag*	71	10.85	3.11				
One-leg stand	68	8.88	1.82	72	8.85	1.86	.02
Walking on toes	68	10.01	2.87	72	9.96	2.89	.02
Jumping on mats	70	10.06	2.49	72	10.04	2.53	.01
Manual dexterity	70	10.93	3.08	72	10.86	3.09	-.02
Aiming & catching	71	10.90	3.05	72	10.93	3.04	.01
Balance	65	9.57	2.98	72	9.62	3.02	.02
Total score	63	11.00	3.45	72	10.80	3.37	-.06

Note. *Pooled descriptives are not available for these items, because they were not imputed. d: dominant; nd: nondominant.

Table B.2

Descriptives on the ZNA-2 and the MABC-2 in 4-year old children

	Original			Pooled			Effect size
	n	*M*	*SD*	*n*	*M*	*SD*	
ZNA-2							
Pegboard (d)	52	.33	1.13	54	.32	1.13	.01
Pegboard (nd)	52	.03	1.16	54	.03	1.17	.00
Bolts (d)	53	.82	.91	54	.83	.92	-.01
Bolts (nd)	54	.70	.93	54	.70	.93	.00
Beads	54	.24	1.07	54	.24	1.07	.00
Repetitive foot (d)*	53	-.32	1.15				
Repetitive foot (nd)*	54	-.25	1.12				
Alternating foot (d)*	52	-.03	1.14				
Alternating foot (nd)*	52	.08	.98				
Repetitive hand (d)*	53	-.60	1.57				
Repetitive hand (nd)*	51	-.67	1.41				
Alternating hand (d)*	51	-.33	1.24				
Alternating hand (nd)*	53	-.27	1.35				
Repetitive fingers (d)*	54	.02	1.43				
Repetitive fingers (nd)*	52	-.12	1.45				

Sequential fingers (d)*	51	.50	1.21				
Sequential fingers (nd)*	50	.31	1.09				
Jumping sideways	51	-.06	.69	54	-.05	.69	-.01
Chair rise	52	-.19	1.32	54	-.15	1.33	-.03
Long jump	50	.30	1.16	54	.24	1.21	.05
Fine motor skills	52	.65	1.06	54	.63	1.05	-.02
Pure motor skills	41	-.08	1.16	54	-.24	.86	-.19
Static balance	45	-.53	.96	54	-.62	1.01	-.09
Dynamic balance	46	.04	.98	54	.03	.98	.00
Total score	28	-.03	1.00	54	-.07	.97	-.04
MABC-2							
Posting coins (d)	53	10.85	2.63	54	10.84	2.63	.00
Posting coins (nd)	53	10.25	2.22	54	10.24	2.22	.00
Beads	53	9.98	2.20	54	9.95	2.22	.01
Drawing trail	53	8.68	2.30	54	8.66	2.31	.01
Catching a bean bag*	53	8.77	2.61				
Throwing a bean bag*	54	9.85	2.65				
One-leg stand	53	8.31	2.53	54	8.26	2.56	.02
Walking on toes	52	9.9	3.27	54	9.87	3.30	.01
Jumping on mats	53	9.62	3.27	54	9.62	3.26	.00
Manual dexterity	53	9.57	2.57	54	9.56	2.55	.00
Aiming & catching	53	9.17	2.38	54	9.17	2.36	.00

Balance	52	9.06	3.15	54	9.08	3.11	.01
Total score	51	9.08	2.35	54	9.11	2.38	.01

Note. *Pooled descriptives are not available for these items, because they were not imputed. d: dominant; nd: nondominant.

Table B.3

Descriptives on the ZNA-2 and the MABC-2 in 5-year old children

	Original			Pooled			Effect size
	n	*M*	*SD*	*n*	*M*	*SD*	
ZNA-2							
Pegboard (d)	42	-.01	.73	43	.01	.75	-.03
Pegboard (nd)	43	.06	1.21				
Bolts (d)	42	-.03	.93	43	-.04	.93	.01
Bolts (nd)	43	-.07	.95				
Beads	43	.19	1.16				
Repetitive foot (d)*	40	.04	1.25				
Repetitive foot (nd)*	40	.13	1.23				
Alternating foot (d)*	42	.07	1.163				
Alternating foot (nd)*	42	.07	.97				
Repetitive hand (d)*	41	-.07	1.77				
Repetitive hand (nd)*	41	-.27	1.52				
Alternating hand (d)*	41	-.20	1.20				
Alternating hand (nd)*	41	.32	1.38				

Repetitive fingers (d)*	41	.03	1.38				
Repetitive fingers (nd)*	40	.07	1.42				
Sequential fingers (d)*	40	.28	1.29				
Sequential fingers (nd)*	39	.33	1.34				
Jump sideways	38	-.02	1.26	43	-.02	1.22	.00
Chair rise	39	-.15	1.31	43	-.05	1.34	-.07
Long jump	41	-.04	1.26	43	-.01	1.27	-.02
Fine motor skills	41	.03	.85	43	.06	.85	.04
Pure motor skills	35	.15	1.39	43	.12	1.00	-.03
Static balance	38	-.90	1.18	43	-.68	1.35	.22
Dynamic balance	33	-.27	1.33	43	-.05	1.33	-.17
Total score	26	-.50	1.07	43	-.20	1.19	.25
MABC-2							
Posting coins (d)	42	10.40	2.96	43	10.39	2.95	.00
Posting coins (nd)	41	10.59	2.33	43	10.54	2.36	.02
Beads	42	9.60	2.86	43	9.58	2.85	.01
Drawing trail	43	8.51	2.33				
Catching a bean bag*	41	8.46	3.18				
Throwing a bean bag*	43	9.84	3.00				
One-leg stand	43	7.38	2.62				
Walking on toes	42	9.12	3.05	43	9.12	3.05	.00
Jumping on mats	43	9.05	3.47				

Manual dexterity	41	9.41	2.46	43	9.38	2.48	-.01
Aiming & catching	41	8.98	3.13	43	9.09	3.12	.04
Balance	42	8.00	2.88	43	7.95	2.87	-.02
Total score	38	8.39	3.25	43	8.42	3.08	.01

Note. *Pooled descriptives are not available for these items, because they were not imputed. d: dominant; nd: nondominant.
